# Evaluation of smoking cessation counseling practices of physicians working in primary health care institutions

**DOI:** 10.55730/1300-0144.5807

**Published:** 2024-01-08

**Authors:** Fatma Nur KARAÇORLU, Edibe PİRİNÇCİ

**Affiliations:** 1 Bulanık District Health Directorate, Muş, Turkiye; 2 Department of Public Health, Faculty of Medicine, Fırat University, Elazığ, Turkiye

**Keywords:** Smoking cessation, primary health care, physician, preventive health care

## Abstract

**Background/aim:**

In this study, we aimed to evaluate the self-reported practice of physicians working in primary health care institutions in Elazığ Province about smoking cessation counseling.

**Materials and methods:**

The population of this cross-sectional study consisted of 262 physicians working in primary health care institutions in Elazığ. We aimed to reach the entire population without using the sample selection method and we successfully reached 95.42% (250 people) of the population. A questionnaire form was used for data collection. The data obtained were evaluated by frequency, percentage, mean ± standard deviation, median and minimum–maximum, and binary logistic regression analysis.

**Results:**

The mean age of the physicians was 40.86 ± 10.58 years and 68.0% of them were male. Among them, 30.4% (n = 76) were current smokers, 17.6% (n = 44) were former smokers and 52.0% (n = 130) were never smokers. The physicians’ frequency of applying the steps of smoking cessation counseling was as follows: 38.8% “Ask”, 81.6% “Advice”, 68.4% “Assess”, 66.8% “Assist”, 31.2% “Arrange”. Additionally, 16.0% of the physicians received smoking cessation counseling training. Those who had not smoked at least 100 cigarettes in their life applied the “Advice” (p = 0.026) step more frequently. Those who received smoking cessation counseling training applied the following steps more frequently: “Ask” (p = 0.024), “Assist” (p = 0.025), and “Arrange” (p = 0.001).

**Conclusion:**

It is seen that the smoking frequency of primary care physicians in Elazığ Province is the same as that of the general population, and the frequency of smoking cessation counseling is far behind the target. Physician population is an important target group that should not be ignored in the fight against smoking. In order to increase the frequency of physicians’ smoking cessation counseling practice, there is a need to increase the number of physicians who receive smoking cessation counseling training.

## 1. Introduction

The tobacco epidemic is one of the biggest public health threats facing the world. According to World Health Organization (WHO) data, there are 1.3 billion tobacco smokers aged 15 and over worldwide^1^. Globally, in 2019, the prevalence of smoking was 29.6% in men and 5.3% in women [1]. According to Türkiye Health Statistics Yearbook 2019, 31.4% of all individuals aged 15 and over in Türkiye were tobacco users (28% regular smokers and 3.4% occasional smokers). Specifically, 44.8% of men and 18.1% of women were tobacco users [2].

Tobacco use stands as the leading cause of preventable disease and death^2^. Annually, 8 million people in the world^1^ and more than 83 thousand people in Türkiye die due to smoking-related causes^3^. Among the diseases caused by tobacco use, cardiovascular diseases, lung cancer, and chronic obstructive pulmonary disease (COPD) come to the fore [3].

In line with the Framework Convention on Tobacco Control, WHO has recommended the M-POWER (monitoring, protect, offer, warn, enforce, raise) policy package to all member countries to guide tobacco control efforts. The “O (offer)” component of this policy package is to express support for those who want to quit smoking^4^. There are structured brief tobacco intervention models available that can guide physicians to talk and advise tobacco users about tobacco use. One of the models is the 5A (Ask, Advise, Assess, Assist, Arrange) model [3, 4].

Physicians play a key role in tobacco control and tobacco addiction treatment. They are expected not only to be role models in tobacco use but also to address tobacco addiction as a part of their standard care practices. It is known that brief advice given by physicians increases the frequency of smoking cessation by 66%, while intensive advice increases it by 84% compared to the absence of any advice [4]. A metaanalysis of 43 studies on smoking cessation counseling reported that minimal counseling (<3 min) increased the frequency of smoking cessation by 30%, low-intensity counseling (3–10 min) by 60%, and high-intensity counseling (>10 min) by 130%^5^. In particular, primary care physicians have a unique position compared to other physicians in helping tobacco users. The reasons for this situation can be listed as follows [4]: 1) Primary care physicians have a long-term and close relationship with the society. 2) Primary care is the primary source of health care, allowing primary health care providers to reach the majority of the population in many countries. 3) Primary care programs are more effective at reaching the poor much than other health programs, and it is even more important to reach the poor, given that it is the poor who smoke the most.

Services are provided through the Smoking Cessation Advice Line and smoking cessation polyclinics for individuals who want to quit using tobacco products in Türkiye^2^. The Smoking Cessation Advice Line receives an average of 4000 calls daily. As of the end of 2018, there were 501 smoking cessation polyclinics in our country. In these polyclinics, between January 1, 2009, and December 31, 2018, these polyclinics conducted 2,381,782 examinations. Since 2010, 949,964 people have benefited from smoking cessation treatment drugs provided free of charge by the Ministry of Health^4^. Considering that 31.4% of the population aged 15 and over in Türkiye are tobacco users^6^, it is evident that the number of operating smoking cessation polyclinics throughout the country is quite low. Due to this insufficiency, it is not possible to provide adequate support to smokers in the country [3]. For this reason, it is of great importance that smoking cessation treatment services are not only provided through specialized polyclinics but also expanded to include primary health care services, particularly^2^ [3].

Based on the reasons that the tobacco epidemic is one of the most important public health threats worldwide and in Türkiye^1^, and considering the vital importance of physicians’ involvement in combating tobacco use, as emphasized in [5], as well as the necessity to expand smoking cessation counseling service not only in smoking cessation polyclinics but also across all health care steps, especially in primary care^2^, this research was planned to be conducted. In this study, we aimed to determine the frequency and related factors of primary care physicians in Elazığ Province to apply each step of the 5A strategy of smoking cessation counseling, the education, knowledge, beliefs, and perceived barriers of physicians about smoking cessation counseling.

## 2. Methods

The data collection phase of this cross-sectional study was carried out in primary health care institutions in the province of Elazığ between March and May, 2021. The population of the study consisted of 262 physicians working in primary care in the province of Elazığ. In the study, it was aimed to reach the entire population without using any sample selection method, and 250 physicians (95.4%) were reached.

For data collection, a questionnaire developed by the researchers by scanning the literature was used. The dependent variable was the frequency of application of the 5A method steps of smoking cessation counseling [3]. There were 5 options in the questionnaire (never/rarely/sometimes/often/always), and 2 categories were created out of 5 options for analysis (never-rarely-sometimes and often-always). Independent variables: sex, age, smoking status, age at onset of smoking, receiving and willingness to receive smoking cessation counseling training outside of undergraduate education, knowledge, beliefs, and perceived barriers to smoking cessation counseling. Smoking status was classified as follows^7^: Current smoker: Individuals who have smoked at least 100 cigarettes in their lifetime and are still current smokers. Former smoker: Individuals who have smoked at least 100 cigarettes in their lifetime but have quit at the time of the interview. Never smoker: Individuals who have never smoked or have smoked fewer than 100 cigarettes in their lifetime. The questionnaire included four options (strongly disagree/disagree/agree/strongly agree) for assessing beliefs and perceived barriers to smoking cessation counseling.

After obtaining the ethics committee approval and institutional permissions for the research, the data collection process was started. The list of names and workplaces of primary care physicians was obtained from the Elazığ Provincial Health Directorate. After the necessary information was given about the study and verbal informed consent was obtained, the participants were asked to fill in the questionnaire. The questionnaire was applied mainly with a printed questionnaire, but an online questionnaire was sent to the physicians working in places that could not be reached due to geographical distances and/or physical access difficulties. Of the physicians included in the study, 187 (74.8%) filled out a printed questionnaire and 63 (25.2%) filled out an online questionnaire. 76.8% (n = 192) of the physicians participating in the study work in a family health center, and 23.2% (n = 58) work in other primary health care institutions.

Data were evaluated using SPSS v. 21.0. Descriptive statistics were presented as frequency (n) and percentage (%) for categorical variables, mean ± standard deviation (Mean ± SD) and/or median and minimum (min)–maximum (max) for continuous variables. Binary logistic regression analysis was performed to determine the effect of the independent variables on the dependent variable of the frequency of application of the 5A steps of smoking cessation counseling. Odds ratio (OR) was given together with 95% confidence interval (CI) as a result of regression analysis. In the regression analysis, unadjusted odds ratio (UOR) values were given in unadjusted models and adjusted odds ratio (AOR) values were given in adjusted models. In statistical analyses, p < 0.05 was accepted as significant.

The ethics committee approval of the study was obtained from the Fırat University Non-Interventional Research Ethics Committee with the letter dated 11.12.2020 and numbered 428932, and the institutional permission was obtained from the Elazığ Provincial Health Directorate with the commission decision dated 14.01.2021.

## 3. Results

The majority of the primary care physicians (68.0%, n = 170) were male, and the mean age of all participants was 40.86 ± 10.58 (min = 24, max = 67, median = 41). 30.4% (n = 76) of the physicians were current smokers, 17.6% (n = 44) were former smokers, and 52.0% (n = 130) were never smokers. The mean age of smoking onset of physicians who smoked was 20.77 ± 4.59 (min = 12, max = 38, median = 20).

**Figure 1:**
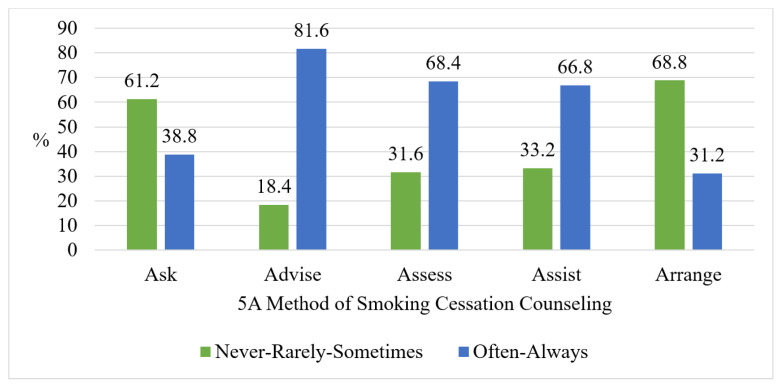
Frequency of physicians applying the 5A method of smoking cessation counseling.

The frequency of application of the 5A approach steps of smoking cessation counseling by physicians is illustrated in Figure 1. Only 3.6% (n = 9) of the physicians answered “never” to the “Ask” step, with no instances of “never” reported for the other steps. Among the physicians who asked about the smoking status of patients, 62.2% (n = 150) stated that they did so only when the patients had smoking-related illnesses. On the other hand, 37.8% (n = 91) reported asking patients about their smoking status even in the absence of smoking-related illnesses. Regarding advising patients to quit smoking, 16% (n = 41) of the participants stated that they only provided such advice when patients had smoking-related illnesses. Conversely, the vast majority (83.6%, n = 209) indicated that they advised patients to quit smoking even when in the absence of any smoking-related illness.

Sixteen percent of (n = 40) of the physicians have received smoking cessation counseling training, and 52.8% (n = 132) of all physicians expressed a desire to receive training. Additionally, 88.0% (n = 220) of the physicians recalled the number of the Smoking Cessation Advice Line correctly, confirming it as 171. The distribution of the answers given by physicians to the knowledge questions about Smoking Cessation Counseling is given in Table 1.

**Figure 2:**
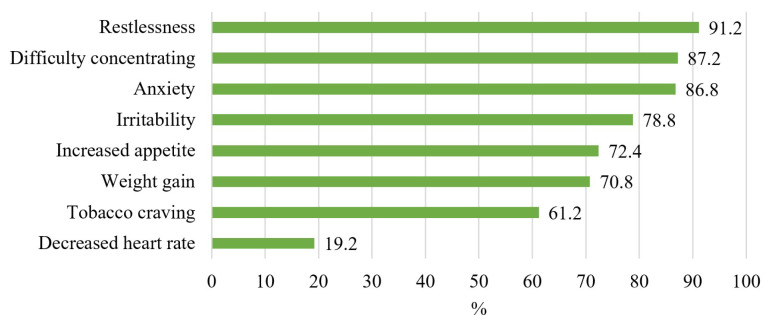
Knowledge on physicians’ symptoms of nicotine withdrawal.

The most marked nicotine withdrawal symptom by physicians was “restlessness” with 91.2% (n = 228), followed by “difficulty concentrating” with 87.2% (n = 218), and “anxiety” with 86.8% (n = 217). The least frequently marked symptom of nicotine withdrawal was “decreased heart rate” with 19.2% (n = 48) (Figure 2).

Table 2 shows the frequency of agreeing/strongly agreeing with the statements used to evaluate the beliefs of primary care physicians about smoking cessation counseling.

Perceived barriers to smoking cessation counseling by physicians are presented in Table 3. The most common barrier among physicians for smoking cessation counseling was “workload intensity” (n = 205, 82.0%). The second most common perceived barrier was physicians feeling that they lacked sufficient information for smoking cessation counseling (n = 178, 71.2%).

Physicians who have not smoked at least 100 cigarettes in their lifetime (never smoker) advised patients to quit smoking more often than smokers who have smoked at least 100 cigarettes (current and former smokers) (UOR: 2.12, CI: 1.10–4.10, p = 0.026). Physicians who received smoking cessation counseling training were more likely to apply the “Ask” (UOR: 2.20, 95% CI: 1.11–4.36, p = 0.024), “Assist” (UOR: 2.67, 95% CI: 1.13–6.34, p = 0.025), and “Arrange” (UOR: 3.36, 95% CI: 1.68–6.73, p = 0.001) steps. Physicians who want to receive smoking cessation counseling training were more likely to apply the “Advise” (UOR: 2.20, 95% CI: 1.14–4.26, p = 0.019) and “Assist” (UOR: 2.21, 95% CI: 1.29–3.77, p = 0.004) steps. As the number of correctly answered questions increased, the frequency of physicians applying the “Assist” step increased (UOR: 1.22, 95% CI: 1.08–1.39, p = 0.002) (Table 4).

Table 5 shows the relationship between lifetime smoking of at least 100 cigarettes and various beliefs and perceived barriers related to smoking cessation counseling. The frequency of agreeing with the belief that medication is helpful for quitting smoking (p = 0.005) and smoking cessation counseling is effective (p = 0.003) was significantly less in those who smoked at least 100 cigarettes in life compared to nonsmokers. The frequency of agreeing in the perceived barrier of not considering smoking cessation counseling as their duty was significantly higher among physicians who have smoked at least 100 cigarettes in their lifetime compared to nonsmokers (p = 0.039)

## 4. Discussion

In our study, the frequency of current smokers was 30.4%, former smokers 17.6%, and never smokers 52.0%. Considering that 31.4% of the population aged 15 and over in Türkiye are current smokers, 14.2% are former smokers, and 54.5% are never smokers^6^, it is seen that the primary care physicians included in the study have a similar smoking frequency as in Türkiye in general. In a study conducted between 2010 and 2011 representing family physicians in Türkiye, current smokers were found to be 34.1%, former smokers 14.8%, and never smokers 51.1% [6]. Similarly, in another study conducted in 2016, current smokers were 30.9%, former smokers 17.5%, and never smokers 51.6% [7]. In our study conducted in 2021, similar frequencies were found in line with the literature. Based on these findings, it is seen that the smoking frequency of primary care physicians in Türkiye has not changed in the last 10 years. When the prevalence of smoking in physician populations worldwide is compared, physicians’ smoking frequency varies from country to country due to differences in the definition of the smoker and the sex and age distribution among physicians [8].

In the current study, the mean age of physicians to start smoking was found to be 20.77 ± 4.59 years. Consistent with the findings of our study, the mean age of onset of smoking among physicians was reported as 21.73 ± 5.04 in a study involving family physicians across Türkiye [6], and 21 ± 4 in a study conducted with physicians working in primary care in Isparta, Türkiye [9]. Unlike these findings, according to the Global Adult Tobacco Survey 2016 Türkiye report, the average age of starting smoking in individuals over the age of 15 in Türkiye was 17.0^8^. It is seen that the average age at which primary care physicians start smoking is higher than that of the general population. Additionally, the age at which physicians begin smoking aligns with the time they were in medical school [9]. In a study conducted with family physicians across Türkiye, it was determined that 55.1% of smokers started smoking during their university years [6]. This can be explained by the fact that nonsmokers in the preuniversity period started to smoke because of the stress they faced when starting the medical [10, 11].

In the current study, 38.8% of the primary care physicians reported asking patients about their cigarette use at each appointment, and 81.6% of them stated that they advised all smokers to quit (Figure 1). According to the Global Adult Tobacco Survey 2016 Türkiye report, 46% of those aged 15 and over who applied to a doctor due to any health problem were asked about their smoking status, and 87.4% of smokers were advised to quit^2^,^8^. It is seen that the findings of our study are compatible with the Global Adult Tobacco Survey 2016 Türkiye report. However, these results fall behind the 2021 targets set in the Türkiye Tobacco Control Strategy Document and Action Plan 2018–2023. According to the plan, these frequencies are expected to be 80% and 95% for the “Ask” and “Advise” steps, respectively^2^. It is also noteworthy that in our study, the “Advise” step of the 5A method of smoking cessation counseling was applied more frequently than the “Ask” step (Figure 1). In similar studies conducted in Türkiye, Argentina, and Syria, it has been reported that the “Advise” step is applied more frequently than the “Ask” step [7, 12–14].

In our study, it was determined that the least applied step (31.2%) of the 5A strategy of smoking cessation counseling was “Arrange” (Figure 1). In a systematic review that included 35 articles examining the smoking cessation counseling practices of primary care physicians, the frequency of physicians applying the 5A steps was determined as follows: Ask 65% (min: 7%, max: 100%), Advice 63% (min: 13%, max: 99%), Assess 36% (min: 11%, max:72%), Assist 44% (min: 2%, max: 98%), and Arrange 22% (min: 2%, max: 54%) [15]. In this review, consistent with the findings of our study, it is seen that “Arrange” was the least applied step of the 5A strategy for smoking cessation counseling. In addition, very wide ranges have been reported for the frequency of application of each step of smoking cessation counseling. The review mentioned that the discrepancy in reported counseling practices could be attributed to variations in the number of questions used to evaluate self-reported counseling across different studies, differences in how the questions are phrased, as well as variations in the number and types of response options provided. It was emphasized that this situation limits the comparability of the results of the studies; therefore, a standardized tool is needed to evaluate the self-reported counseling practice [15]. Although tools have been developed to evaluate smoking cessation counseling in relation to the 5A strategy, further evaluation of these tools is necessary. Most of these instruments are unpublished, and there is often a lack of reliability and validity data available for them [16].

In the current study, 62.2% of the physicians included in the study did not ask about cigarette use when the patients did not have a smoking-related illness, and 16.4% did not advise patients to quit smoking in the absence of a smoking-related illness. However, in the Türkiye Tobacco Control Strategy Document and Action Plan 2018–2023, it is recommended that a brief clinical interview be applied by health professionals in all encounters to all individuals aged 15 and over who apply to health institutions and organizations due to any health problem^2^. Although the brief clinical interview is defined by WHO as a 3–10 min interview [4], a 3–10 min interview is of course not realistic and impractical in busy environments where doctors usually only have 5–10 min for each patient. However, it is known that even a very brief smoking cessation intervention of about 30 sec during regular medical patient interviews by doctors increases the smoking cessation of smokers by 14% to 42% [17].

In the current study, physicians were asked whether they had received any training other than undergraduate education in order to provide smoking cessation counseling to patients, and only 16.0% stated that they had received any training. According to Türkiye Tobacco Control Strategy Document and Action Plan 2018–2023, within the scope of strengthening smoking cessation services, it is aimed to ensure that family physicians complete the smoking cessation treatment distance education module and the frequency of trained family physicians will be 100% in 2021^2^. In our study, it is seen that the frequency of physicians receiving smoking cessation counseling training fell far behind this target. In studies conducted in Türkiye and Palestine, it was reported that very few primary care physicians received smoking cessation counseling training [9, 14, 18].

Among the knowledge questions asked about smoking cessation counseling to primary care physicians; The Smoking Cessation Advice Line number, the questions determining the level of addiction of the smoker (Table 1), and the symptoms of nicotine withdrawal (Figure 2) were known to the vast majority of the physicians. However, the frequency of giving correct answers to questions about pharmacological treatment in smoking cessation counseling was quite low compared to other questions (Table 1). In a similar study conducted in Saudi Arabia, primary care physicians were observed to provide correct answers to the questions about pharmacological treatment in smoking cessation counseling the least frequently [19]. For this reason, smoking cessation counseling trainings provided to physicians should prioritize pharmacological treatment over other topics.

Nine out of ten physicians participating in the study believe that smoking cessation pharmacological treatment and counseling is beneficial and effective. In addition, a significant number of physicians agreed that physician advice increases the probability of individuals to quit smoking (Table 2). In studies conducted in Türkiye, China, and Saudi Arabia, it was found that the majority of physicians found smoking cessation counseling effective [7, 20–22] and agreed that the advice of a health care professional increases the probability of quitting smoking [9, 19, 22, 23].

The vast majority of physicians agreed with the following statement: “Physicians should receive special training in smoking cessation counseling.” (Table 2). This finding is consistent with the literature [9, 20, 22].

Consistent with other studies, the belief that smoking physicians may be less likely to advise people to quit smoking was one of the least frequently agreed beliefs about smoking cessation counseling questioned (Table 2) [9, 19, 22, 23].

The least frequent belief regarding smoking cessation counseling was that primary health care facilities are a suitable place to provide smoking cessation counseling (Table 2). Contrary to the opinions of primary care physicians, primary care is a suitable environment for smoking cessation counseling for various reasons [15]. First, family medicine normally constitutes the first point of medical contact with the health system^9^. Second, family physicians meet with individuals older than 18 years of age in their registered population at least once a year. According to the Directive on Family Medicine Screening and Follow-up Coefficient, family physicians should perform routine follow-up at least once a year in healthy adults older than 18 years of age in their registered population, and the frequency of follow-up increases in individuals with chronic diseases^10^. Third, regular personal contact in primary care builds trust between physicians and patients and facilitates the provision of quit advice [24]. Fourth, face-to-face contact allows for individual smoking cessation advice [25]. Finally, the fact that 35% of applications to physicians were reported to primary health care institutions, according to the Türkiye 2019 Health Statistics Yearbook [2], shows that primary care has an important place in health service delivery. To summarize, it is a significant opportunity to provide smoking cessation counseling when patients apply to primary health care institutions, because family medicine is the first point of contact for individuals, regular follow-up is carried out at least once a year, primary care plays an important role in the delivery of health services. In addition, according to Türkiye Tobacco Control Strategy Document and Action Plan 2018–2023, there are some targets within the scope of strengthening smoking cessation services. It is aimed to expand smoking cessation polyclinics in institutions providing primary health care services other than family health centers in order to increase the number of units providing smoking cessation services. It is aimed to provide smoking cessation services in 90% of family health centers in 2021^2^.

In our study, 68.4% of the physicians stated that smoking cessation counseling is very time-consuming (Table 3). According to the results of a systematic review of 19 studies to investigate perceived barriers to smoking cessation counseling by primary care physicians, the most frequently cited perceived barrier was that smoking cessation counseling was too time-consuming, with 42% (95% CI: 29.0–55.3) [26]. However, even a very short 30-sec smoking cessation intervention given by the physician is effective in increasing the frequency of smoking cessation of patients [17].

More than half (58.8%) of primary care physicians stated that it is difficult to obtain smoking cessation drugs (Table 3). According to Türkiye Anti-Tobacco Annual Report 2018, 949,964 people have been provided with drugs to help them quit smoking since 2010, within the scope of smoking cessation counseling services^4^. Considering that there are approximately 20 million people over the age of 15 who use tobacco and tobacco products in Türkiye, and 17 million of them use it every day^6^ [2], it can be said that sufficient number of drugs cannot be provided within the scope of smoking cessation counseling.

The vast majority of physicians did not agree with the idea that smoking cessation counseling is a low-priority issue both for patients (61.6%) and within the work routine (62.0%) (Table 3). Due to the fact that the tobacco epidemic is one of the biggest public health threats faced by the world^1^ and that tobacco use is among the leading causes of preventable diseases and deaths^2^, tobacco and cigarette use maintains its importance and smoking cessation counseling continues to be a priority in the provision of health services.

In the current study, the perceived barrier that was mentioned least frequently was the perception that giving smoking cessation counseling without asking the patient would violate the patient’s private life (Table 3). In a systematic review, this perceived barrier was reported as 5% (95% CI: 1.9–8.3) [26].

In our study, the smoking status of the physicians and the frequency of advising the patients to quit smoking were found to be significantly related (Table 4). Studies conducted in Türkiye, Argentina, and Saudi Arabia have also reported that nonsmoker physicians provide smoking cessation counseling more frequently [9, 12, 27, 28]. The reason why nonsmoker physicians advise patients to quit smoking more frequently than smoker physicians may be that nonsmoker physicians are more likely to believe that smoking cessation medication and smoking cessation counseling are effective (Table 5). In addition, smoking physicians are more likely to agree that smoking cessation counseling is not their duty than nonsmoker physicians (Table 5). This may be related to the fact that smoker physicians advise patients to quit smoking less frequently than nonsmokers.

In our study, having received smoking cessation counseling training was found to be significantly associated with 3 of the 5 steps (Ask, Assist, and Arrange) (Table 4). In an intervention study involving primary care physicians, the physicians in the experimental group were trained on smoking cessation counseling, while those in the control group were not. The results of the intervention were evaluated by applying a questionnaire to the patients of the physicians. According to the results of the study, individuals who met with the trained physicians received smoking cessation counseling at a higher rate and these people also quit smoking at a higher rate. This shows that providing smoking cessation counseling training to primary care physicians increases both the frequency and effectiveness of smoking cessation interventions [29]. In addition, it has been shown in many cross-sectional studies in the literature that physicians who have received training in smoking cessation counseling provide smoking cessation counseling more frequently [7, 9, 18, 20, 27, 30].

This study has some limitations. First of all, the inability to use a standard measurement tool to evaluate the smoking cessation counseling practices of physicians limited the comparability of the findings of the current study with other studies in the literature. Secondly, the fact that the data on the practice of the participants were not collected by the researchers by observation but by the participants by filling in the questionnaires may have caused the results to be overreported. Third, since the universe of the research is spread over a wide geography, the answers of the participants who filled in online may have made a difference compared to those who filled out the printed questionnaires. Fourth, the results of this study are limited to primary care physicians in Elazığ Province and cannot be generalized to primary care physicians working in different geographies. Finally, the cross-sectional design of this study precludes us from making causal inferences. More research is needed to find causal relationships between variables.

As a result, it is seen that the smoking frequency of primary care physicians in Elazığ Province is the same as the general population, and the frequency of smoking cessation counseling is far behind the target. Since physicians are role models for the society with their health behaviors, first of all, the frequency of smoking in the physician population should be examined and attempts should be made to reduce it. In order to increase the frequency of smoking cessation counseling, there is a need to increase the number of physicians who receive smoking cessation counseling training and to strengthen the positive beliefs of physicians about smoking cessation counseling with these trainings.

## Figures and Tables

**Table 1:** Knowledge on physicians’ smoking cessation counseling.

True	n	%
If a smoker is very ill or spends most of the day in bed but still smokes, it means he or she is more addicted.	206	82.4
If the first cigarette in the morning is more delicious for the smoker, this means that the person is more addicted.	203	81.2
The most common relapse after smoking cessation occurs in the first month and especially in the first week.	187	74.8
Nicotine replacement therapy should not be given to pregnant and lactating women.	138	55.2
One of the drugs used in smoking cessation treatment is varenicline.	128	51.2
**False**		
A single interview is sufficient for smoking cessation counseling.	232	92.8
There is no relationship between the amount of cigarettes smoked daily by a cigarette user and the level of addiction.	214	85.6
The later the smoker smokes her/his first cigarette in the morning, the more addicted she/he is.	186	74.4
Bupropion is not used for smoking cessation therapy.	147	55.8
Those who smoke larger amounts of cigarettes in the evening are more likely to be addicted to cigarettes.	101	40.4
Nicotine replacement therapy is administered to patients with low levels of nicotine addiction.	78	31.2

**Table 2:** Beliefs on physicians’ smoking cessation counseling.

Beliefs	Agree/strongly agree	
	n	%
Medication is helpful for quitting smoking.	226	90.4
Smoking cessation counseling is effective.	225	90.0
Physicians should routinely advise their patients who smoke to quit smoking.	217	86.8
Physicians should receive special training on smoking cessation counseling.	207	82.8
Physicians should routinely ask about their patients’ smoking habits.	198	79.2
It is an important opportunity for smoking cessation counseling when patients apply to primary health care institutions.	189	75.6
If the physician advises the patient to quit smoking, the probability of the patient to quit smoking increases.	187	74.8
Physicians who smoke are less likely to advise people to quit smoking.	160	64.0
Primary health care institutions are a suitable place for smoking cessation counseling.	156	62.4

**Table 3:** Perceived barriers to smoking cessation counseling by physicians.

Perceived barriers	Agree/strongly agree	
	n	%
It is difficult to give smoking cessation counseling due to the heavy workload.	205	82.0
I do not have enough information about smoking cessation counseling.	178	71.2
Smoking cessation counseling takes a lot of time.	171	68.4
Patients are resistant to talking about smoking.	163	65.2
It is difficult to obtain smoking cessation medications for patients.	147	58.8
I do not feel confident about smoking cessation counseling.	122	48.8
I do not think that smoking cessation counseling is my duty.	98	39.2
Smoking cessation counseling is a low priority for patients.	96	38.4
Smoking cessation counseling is a low priority issue in the work routine.	95	38.0
Questioning the smoking status of patients even though they do not request smoking cessation counseling is a violation of patients’ private lives.	48	19.2

**Table 4:** Binary logistic regression analysis predicting physicians’ frequency of applying the 5A methods of smoking cessation counseling, odds ratio (95% confidence interval).

Variables	Ask		Advise		Assess		Assist		Arrange	
	Unadjusted	Adjusted*	Unadjusted	Adjusted*	Unadjusted	Adjusted*	Unadjusted	Adjusted*	Unadjusted	Adjusted*
At least 100 cigarette smoking status in life										
Yes	1	1	1	1	1	1	1	1	1	1
No	1.04 (0.62–1.73)	1.11 (0.62–2.01)	2.12 (1.10–4.10)	2.28 (1.05–4.95)	1.17 (0.68–1.99)	1.26 (0.67–2.34)	1.01 (0.60–1.71)	1.43 (0.74–2.73)	1.20 (0.70–2.05)	1.33 (0.70–2.53)
p	0.884	0.718	0.026	0.036	0.571	0.473	0.966	0.284	0.505	0.381
Getting smoking cessation counseling training										
Yes	2.20 (1.11–4.36)	1.98 (0.98–4.00)	1.70 (0.63–4.60)	1.20 (0.42–3.41)	2.04 (0.89–4.67)	1.65 (0.70–3.88)	2.67 (1.13–6.34)	1.80 (0.72–4.47)	3.36 (1.68–6.73)	3.00 (1.45–6.19)
No	1	1	1	1	1	1	1	1	1	1
p	0.024	0.059	0.298	0.732	0.090	0.253	0.025	0.205	0.001	0.003
Willingness to receive smoking cessation counseling training										
Yes	0.99 (0.59–1.64)	0.92 (0.54–1.56)	2.20 (1.14–4.26)	2.22 (1.11–4.46)	1.65 (0.96–2.82)	1.78 (1.02–3.14)	2.21 (1.29–3.77)	2.55 (1.42–4.59)	1.06 (0.62–1.82)	1.07 (0.60–1.90)
No	1	1	1	1	1	1	1	1	1	1
p	0.955	0.758	0.019	0.025	0.068	0.044	0.004	0.002	0.823	0.814
Number of questions answered correctly	1.13 (1.00–1.28)	1.11 (0.97–1.26)	1.12 (0.97–1.29)	1.15 (0.98–1.35)	1.07 (0.94–1.21)	1.07 (0.94–1.22)	1.22 (1.08–1.39)	1.25 (1.09–1.44)	1.08 (0.95–1.22)	1.06 (0.92–1.22)
p	0.051	0.129	0.124	0.090	0.292	0.306	0.002	0.002	0.266	0.404

* The model was adjusted by age and sex

**Table 5:** Relationship between at least 100 cigarette smoking status in life and some beliefs and perceived barriers related to smoking cessation counseling

	At least 100 cigarette smoking status in life, n (%)*		p
	Yes	No	
Medication is helpful for quitting smoking.			**0.005**
Agree/strongly agree	102 (85.0)	124 (95.4)	
Disagree/ strongly disagree	18 (15.0)	6 (4.6)	
Smoking cessation counseling is effective.			**0.003**
Agree/strongly agree	101 (84.2)	124 (95.4)	
Disagree/ strongly disagree	19 (15.8)	6 (4.6)	
I do not think that smoking cessation counseling is my duty.			**0.039**
Agree/strongly agree	55 (45.8)	43 (33.1)	
Disagree/ strongly disagree	65 (54.2)	87 (66.9)	

* Column percent

## Footnotes

1 World Health Organization (2021). Tobacco [online]. Website https://www.who.int/health-topics/tobacco#tab=tab_1 [accessed 3 August 2023].

2 Türkiye Cumhuriyeti Sağlık Bakanlığı (2018). Tütün Kontrolü Strateji Belgesi ve Eylem Planı 2018-2023 [online]. Website https://dosyaism.saglik.gov.tr/Eklenti/41034/0/tutunkontrolustratejibelgesiveeylemplanipdf.pdf [accessed 3 August 2023].

3 British Broadcasting Corporation (BBC) (2018). Dünya Sigarayı Bırakma Günü’nde Grafiklerle Türkiye [online]. Website https://www.bbc.com/turkce/haberler-turkiye-40092125 [accessed 3 August 2023].

4 Türkiye Cumhuriyeti Sağlık Bakanlığı (2018). Tütünle Mücadele Faaliyet Raporu, 2018 [online]. Website https://hsgm.saglik.gov.tr/depo/birimler/tutun-ve-madde-bagimliligi-ile-mucadele-db/dokumanlar/Raporlar/Tutun_Faaliyet_Raporu_2018_20.08.2019.pdf [accessed 3 August 2023].

5 Fiore MC, Jaén CR, Baker TB, Bailey WC, Benowitz NL et al. (2008). Treating Tobacco Use and Dependence: 2008 Update [online]. Website https://www.ncbi.nlm.nih.gov/books/NBK63952/ [accessed 3 August 2023].

6 Türkiye İstatistik Kurumu (TÜİK) (2019). Türkiye Sağlık Araştırması Raporu, 2019 [online]. Website https://data.tuik.gov.tr/Bulten/Index?p=Turkiye-Saglik-Arastirmasi-2019-33661 [accessed 3 August 2023].

7 Centers for Disease Control and Prevention (CDC) (2017). Adult Tobacco Use Information [online]. Website https://www.cdc.gov/nchs/nhis/tobacco/tobacco_glossary.htm#:~:text=Every%20day%20smoker%3A%20An%20adult,at%20the%20time%20of%20interview [accessed 3 August 2023].

8 World Health Organization (2016). Global Adult Tobacco Survey, Fact Sheet, Turkey 2016 [online]. Website https://extranet.who.int/ncdsmicrodata/index.php/catalog/872/related-materials [accessed 3 August 2023].

9 Türkiye Aile Hekimleri Uzmanlık Derneği (TAHUD) (2011). Aile Hekimliği Avrupa Tanımı [online]. Website https://www.tahud.org.tr/file/96f3dfc7-3961-4272-b5b7-e6cbbd7580ef/AH_AVRUPA_TANIMI-2.pdf [accessed 3 August 2023].

10 Türkiye Cumhuriyeti Sağlık Bakanlığı (2021). Aile Hekimliği Tarama ve Takip Katsayısına İlişkin Yönerge [online]. Website https://hsgm.saglik.gov.tr/depo/Mevzuat/Yonergeler/Aile_Hekimligi_Tarama_ve_Takip_Katsayisina_Iliskin_Yonerge.pdf [accessed 3 August 2023].

## References

[1] World Health Organization WHO Report on the Global Tobacco Epidemic 2021: Addressing New and Emerging Products 1st ed Geneva, Switzerland WHO Press 2021

[2] The Ministry of Health of Türkiye Health Statistics Yearbook 2019 1st ed Ankara, Türkiye Republic of Türkiye Ministry of Health General Directorate of Health Information Systems 2021

[3] Türkiye Cumhuriyeti Sağlık Bakanlığı Tütün Bağımlılığı ile Mücadele El Kitabı (Hekimler İçin). 1. Baskı Ankara, Türkiye Anıl Matbaacılık 2010 (in Turkish)

[4] World Health Organization Strengthening Health Systems for Treating Tobacco Dependence in Primary Care - Part III Training for Primary Care Providers: Brief Tobacco Interventions 1st ed Geneva, Switzerland WHO Press 2013

[5] World Health Organization Framework Convention on Tobacco Control 1st ed Geneva, Switzerland WHO Press 2003

[6] Baltaci D Bahcebasi T Aydin LY Ozturk S Set T Evaluation of smoking habits among Turkish family physicians Toxicology and Industrial Health 2014 30 1 3 11 10.1177/0748233712448113 22627461

[7] Cerci C Oksuz E Sozen F Cetinel Y Ogus E Smoking prevalence among primary care physicians in Turkey and their knowledge, attitudes, and behaviors about smoking cessation treatment World Journal of Pharmaceutical Research 2020 9 8 30 10.20959/wjpr20208-18128

[8] Borgan SM Jassim G Marhoon ZA Almuqamam MA Ebrahim MA Prevalence of tobacco smoking among health-care physicians in Bahrain BMC Public Health 2014 14 1 931 10.1186/1471-2458-14-931 25200373 PMC4165905

[9] Gokirmak M Ozturk O Bircan A Akkaya A The attitude toward tobacco dependence and barriers to discussing smoking cessation: a survey among Turkish general practitioners International Journal of Public Health 2010 55 3 177 183 10.1007/s00038-009-0109-8 20013142

[10] Baştürk M Koç E Sözmen K Arslan M Albaş S Smoking status, anxiety levels and attitudes regarding the law no 4207 of first and sixth class medical faculty students Konuralp Tıp Dergisi 2018 10 3 282 288 (in Turkish). 10.18521/ktd.337562

[11] Karakaş E Zümbül A Balatacı T Durusoy R Yararbaş G Smoking status of medical students at Ege University: a cross-sectional survey of 1040 students in 2018 Tobacco Induced Diseases 2018 16 Suppl 3 A77 10.18332/tid/94780

[12] Morello P Linetzky B Kaplan J Knowledge, attitudes, and practices of Argentine pediatricians regarding second hand smoke exposure in children Archivos Argentinos de Pediatría 2010 108 318 324 10.1590/S0325-00752010000400005 20672189

[13] Asfar T Al-Ali R Ward KD Weg MWV Maziak W Are primary health care providers prepared to implement an anti-smoking program in Syria? Patient Education and Counseling 2011 85 2 201 205 10.1016/j.pec.2010.11.011 21168300 PMC3074023

[14] Üçer H Ersoy Ö Kahraman H Knowledge, attitudes and practices of family physicians about smoking cessation Turkish Journal of Family Practice 2014 18 2 58 62 (in Turkish). 10.2399/tahd.14.00058

[15] Bartsch A-L Härter M Niedrich J Brütt AL Buchholz A A systematic literature review of self-reported smoking cessation counseling by primary care physicians PLOS ONE 2016 11 12 e0168482 10.1371/journal.pone.0168482 28002498 PMC5176294

[16] Glasgow RE Assessing delivery of the five ‘As’ for patient-centered counseling Health Promotion International 2006 21 3 245 255 10.1093/heapro/dal017 16751630

[17] Cheung YTD Jiang N Jiang CQ Zhuang RS Gao WH Physicians’ very brief (30-sec) intervention for smoking cessation on 13 671 smokers in China: a pragmatic randomized controlled trial Addiction (Abingdon, England) 2021 116 5 1172 1185 10.1111/add.15262 32918512 PMC8246886

[18] Maraqa B Nazzal Z Jabareen J Al-Shakhrah K Determinants of smoking cessation counseling favorable practice for primary care physicians: a cross-sectional study from Palestine Journal of Family Medicine And Primary Care 2021 10 3 1275 1281 10.4103/jfmpc.jfmpc_1456_20 PMC8140223 34041165

[19] Alrufaidy A Alsubhi O Khalifa R Bakarman M Knowledge, attitude and barriers regarding smoking cessation counseling among primary health care physicians in Jeddah, 2019 The Journal of Community Health Management 2020 7 3 13 10.18231/j.jchm.2020.002

[20] AlAteeq M Alrashoud AM Khair M Salam M Smoking cessation advice: the self-reported attitudes and practice of primary health care physicians in a military community, central Saudi Arabia Patient Preference and Adherence 2016 10 651 658 10.2147/ppa.S103010 27175065 PMC4854249

[21] Huang K Abdullah AS Huo H Liao J Yang L Chinese pediatrician attitudes and practices regarding child exposure to secondhand smoke (SHS) and clinical efforts against SHS exposure International Journal of Environmental Research and Public Health 2015 12 5 5013 5025 10.3390/ijerph120505013 26006117 PMC4454951

[22] Sonmez CI Aydin LY Turker Y Baltaci D Dikici S Comparison of smoking habits, knowledge, attitudes and tobacco control interventions between primary care physicians and nurses Tobacco Induced Diseases 2015 13 37 10.1186/s12971-015-0062-7 26566385 PMC4642762

[23] Al-Jdani S Mashabi S Alsaywid B Zahrani A Smoking cessation counseling: knowledge, attitude and practices of primary healthcare providers at National Guard Primary Healthcare Centers, Western Region, Saudi Arabia Journal of Family & Community Medicine 2018 25 175 182 10.4103/jfcm.JFCM_142_17 30220847 PMC6130158

[24] Ulbricht S Meyer C Schumann A Rumpf HJ Hapke U Provision of smoking cessation counseling by general practitioners assisted by training and screening procedure Patient Education and Counseling 2006 63 1–2 232 238 10.1016/j.pec.2005.11.005 16531000

[25] Hoch E Muehlig S Höfler M Lieb R Wittchen HU How prevalent is smoking and nicotine dependence in primary care in Germany? Addiction (Abingdon, England) 2004 99 12 1586 1598 10.1111/j.1360-0443.2004.00887.x 15585050

[26] Vogt F Hall S Marteau TM General practitioners’ and family physicians’ negative beliefs and attitudes towards discussing smoking cessation with patients: a systematic review Addiction (Abingdon, England) 2005 100 10 1423 1431 10.1111/j.1360-0443.2005.01221.x 16185204

[27] Mejia R Martinez VG Gregorich SE Pérez-Stable EJ Physician counseling of pregnant women about active and secondhand smoking in Argentina Acta Obstetricia Et Gynecologica Scandinavica 2010 89 4 490 495 10.3109/00016341003739567 20367427 PMC3158573

[28] Al-Hagabani MA Khan MS Al-Hazmi AM Shaher BM El-Fahel AO Smoking behavior of primary care physicians and its effect on their smoking counseling practice Journal Of Family Medicine and Primary Care 2020 9 2 1053 1057 10.4103/jfmpc.jfmpc_894_19 PMC7114030 32318466

[29] Goldstein MG Niaura R Willey C Kazura A Rakowski W An academic detailing intervention to disseminate physician-delivered smoking cessation counseling: smoking cessation outcomes of the Physicians Counseling Smokers Project Preventive Medicine 2003 36 2 185 196 10.1016/s0091-7435(02)00018-x 12590994

[30] Erdem-Demirezen F Evaluation of knowledge, attitude and behavior of family medicine assistants in Ankara province on smoking cessation treatment Specialization Thesis in Medicine University of Health Sciences Ankara 2021 (in Turkish)

